# Development of an IEEE 1451 Plug-and-Play Module for Smart Transducers in Industrial Environments

**DOI:** 10.3390/s22207880

**Published:** 2022-10-17

**Authors:** João Pinheiro, Diogo Oliveira, Luís Neto, Vítor H. Pinto, Gil Gonçalves

**Affiliations:** 1 FEUP—Faculty of Engineering, University of Porto, Rua Dr. Roberto Frias, 4200-465 Porto, Portugal; 2 SYSTEC (DIGI2)—Research Center for Systems and Technologies (Digital and Intelligent Industry Lab), 4200-265 Porto, Portugal

**Keywords:** IEEE 1451, smart transducers, smart sensors, distributed transducer systems, distributed sensor systems

## Abstract

The use of Sensors and Actuators is ubiquitous in an industrial environment. The advent of the Industrial Internet-of-Things (IIoT) and the 4th industrial revolution demands new, more intelligent and more efficient ways to be able to connect, read and control transducers at the plant floor level. Newer control and data science techniques also largely benefit from structured information endpoints available at the edge of the network. The IEEE 1451 standard presents architecture and methodology to solve these problems with the usage of smart transducers, introducing into edge devices concepts such as self-identification and standardization of data communication. In this work, a transducer interface module is developed using the IEEE 1451 standard focused on flexibility, ease of integration and plug-and-play features. Furthermore, a system architecture, based on IEEE 1451.0 is presented, and development and implementation features are explained. This system is then released as an open-source platform to help and motivate the usage of smart transducer systems. At last, the system is tested, results are collected, and a methodology and metrics are defined for comparison between different smart transducer systems.

## 1. Introduction

Sensing and actuation define the interface between a physical process and control engineering. Being capable of precisely collecting information from a process (Sensing) and precisely inputting physical quantities into a process (Actuation) has a definite impact on the control methods used in manufacturing systems. This allows for higher precision techniques and better control over the variables of a system. Control engineering has evolved over the years, providing the industry with more and better solutions to control processes from this standpoint. With every iteration of control systems, the number of sensors and actuators in a loop tends to increase [1]. The more information a controller can collect and directly influence the better and more elaborate its process decision tends to be. Sensors and actuators have become better over the years, with many of them already containing microcontrollers inside although the way to insert them into a system is, many times, still antiquated.

A problem then arises: how to easily attach transducers (sensors and actuators) to a control loop while enabling them with smart features, the main question this work verses on. Another problem stands in the time it takes for someone upgrading a system to integrate a new transducer into an existing system, another problem this work pursues.

**Figure 1:**
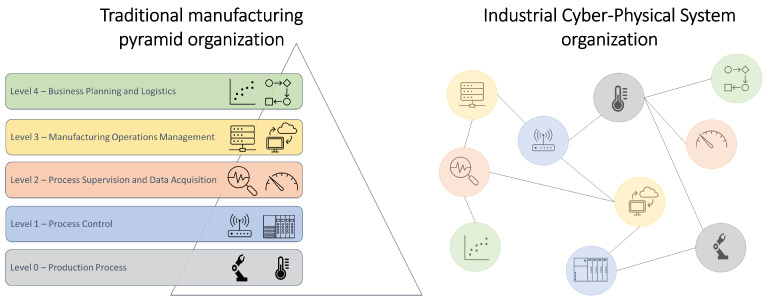
Edge-oriented automation architecture.

Cyber-Physical Systems (CPS) (also known as the drivers of Industry 4.0) introduce the distribution of processing over the network (see Figure 1). In the manufacturing industry, the processing over a network requires the utilization of processing-capable components over the shop floor, which are denominated as edge components, as can be seen in the figure. Providing the loop with edge computing components capable of processing data into information and communicating it over the network can be immensely advantageous:

- The control loop can be distributed over edge-oriented nodes;
- The sensing and actuation can now be provided as a service from the node which drives the transducer;
- Information flows over the network instead of data;
- Multiple systems (Manufacturing execution systems (MES), Enterprise Resource Planning (ERP) systems, Condition Monitoring Systems) can obtain and provide information directly to the control loop components.

Building upon distributed systems communications and utilizing the ability to convey information over the network, information about the transducer itself can also be carried over. An IEEE 1451 smart transducer should be able to identify itself, transmit what it is capable of (e.g., sense, move, calibrate), its manufacturing characteristics (e.g., precision, accuracy, range of movement), and how it will communicate information. Using self-identification, self-calibration, and standardizing of communications, smart transducer networks can be created. Implementing a control loop as a network of smart transducers largely resembles an industrial internet-of-things network, using multiple nodes cooperating towards a goal: better and more integrated manufacturing [2].

Industry 4.0 systems need ubiquitous usage of data in order to develop what are considered to be intelligent systems. The newest trends in research (machine learning techniques (deep learning, neural networks) and big data techniques) require the capability to sense data from machines, manufacturing systems, tools, and even operators. The use of Industry 4.0 manufacturing architectures, such as the Reference Architecture Model for Industry 4.0 (RAMI 4.0) [3], motivate this newest research trend while aiming to improve and upgrade manufacturing processes through more connected and intelligent shop floors.

At the same time, the current production lines are filled with expensive, precise, perfectly functional sensors and actuators, but which are not equipped with the communication and data-handling capabilities newer technologies need. Throwing these transducers away would not be economical nor ecologically desired. Smart Transducers based on IEEE 1451 help solve this problem, facilitating the process of introduction and upgrade of transducers into applications through plug-and-play capabilities.

From the research conducted, there is not, to the authors’ knowledge, an open-source tool that can help develop smart transducers in order to facilitate such said integration.

The main objective of this work is to develop an open-source platform for converting normal sensors and actuators into Smart Transducers based on IEEE 1451 [4,5,6,7,8,9,10]. It shall be capable of interfacing with transducers to collect and deploy data, convert it to information and communicate it to a network in industrial scenarios.

## 2. Background and Bibliographic Review

The topic of sensorization and actuation is one of the most studied areas in electrical engineering. Physical technologies can only go so far as the ability to probe data and manipulate data goes. At an industrial level, sensors and actuators are ubiquitous. Process control depends almost entirely on the ability to probe the status of one ongoing process, decide the course of action using the acquired data, and apply the decision to the process in order to steer it accordingly.

Traditionally, industrial control solutions have been presented as centralized solutions around programmable logic controllers (PLCs) [11]. The development of more complex and computationally heavier control techniques moved the control paradigm into distributed systems in order to conjoin efforts between sparsing processing nodes. This transition has led to the advent of cyber-physical systems which are shaping a new industrial revolution into Industry 4.0 [12].

As important as sensing and actuating is, the ability to receive the probed data and define the physical quantity is something that needs to be actuated. Traditionally, sensors and actuators were considered to be the endpoints of centralized systems. One sensor or one actuator belonged to a control loop and was isolated from the network, i.e., there was no direct access to sensors or actuators. A sensor was seen as one direct wire from which an acquisition card connected to a PLC and probed data. Smart sensors come into play in a conjoined effort to change this paradigm.

In order to fully comprehend the motivation behind the creation of the IEEE 1451 standard, a technical overview of the standard was made.

### 2.1. Technical Overview of IEEE 1451

IEEE 1451 presents the concept of an IEEE 1451-compliant smart transducer as a smart transducer that has more functionalities than those needed to generate a representation of a sensed or controlled quantity. A compliant transducer should provide functions such as self-identification, self-description, self-diagnosis, self-calibration, location-awareness, time-awareness, data processing, reasoning, data fusion, alert notification (report signal), standard-based data formats, and communication protocols [13]. These functions allow the transducer to behave in a plug-and-play fashion. Once connected to the network, the transducer should be capable of identifying itself and presenting its capabilities to other nodes. This ability is provided via a Transducer Electronic DataSheet (TEDS). The TEDS data structure contains information about a transducer much like an ID card, storing information such as manufacturer data and characteristics, measurement range, accuracy, and calibration data. This information is then provided when the handshake between the transducer and other nodes occurs and communication is set.

IEEE 1451 also defines the architecture for the smart transducer. This architecture is divided into two main modules: the Network Capable Application Processor (NCAP) and the Transducer Interface Module (TIM). The communication between the NCAP and the TIM is made via a Transducer Independent Interface (TII) and between the NCAP and the network via the Network Interface(NI). The specific details and functions of each block are specified below.

#### 2.1.1. NCAP-Network Capable Application Processor

The NCAP is responsible for providing smart transducers with higher-level application processing and general application networking capabilities. It is responsible for the creation and consumption of every piece of information from and to the smart transducer. It behaves like a network node and provides the network with services such as ‘read the sensor X’s value’, ‘move the actuator Y 10mm’ or ‘get TEDS’.

#### 2.1.2. TIM-Transducer Interface Module

The TIM (Transducer Interface Module (TIM) and Smart Transducer Interface Module (STIM) are used interchangeably in literature) is responsible for the interface between the sensor/actuator and the NCAP. Its main function is to sample the data sensed or generate the output needed for the actuation. The TIM is also in charge of storing the TEDS and providing it to the NCAP when required. As the TIM interfaces directly with analog signals from and to the transducer, it must contain a way to convert digital to analog values. ADCs, DACs, and Digital I/O are the ways to do this. Being able to create different TIMs custom designed for each application gives the engineering teams a very powerful tool to create smart transducers from scratch. Smart transducers have few limits other than physical ones. The only requirement is that the TIM’s TEDS reports correctly what is present in the system. Once the TEDS is communicated to the network, the data sent by the smart transducer is automatically contextualized and becomes information ready to be processed by other CPS nodes. One TIM can hold information from up to 255 sensors or actuators.

#### 2.1.3. TII-Transducer Independent Interface

The TII defines the interface between the NCAP and the TIM. This interface is responsible for conveying the data collected by the TIM to be processed by the NCAP and the actions received by the NCAP to be carried out by the TIM. The IEEE 1451 standard provides various possibilities for the TII implementation, allowing for vast use cases of these interfaces. In [13], TII is presented as: an SPI interface (IEEE 1451.2); a multi-drop communication transducer network (IEEE 1451.3); a wireless interface (e.g., 802.11-Wifi, 802.15.1-Bluetooth, 802.15.4 (Zigbee), 6LowPAN) (IEEE 1451.5); a high-speed CANopen network (P1451.6); and an RFID system (IEEE 1451.7).

#### 2.1.4. NI-Network Interface

The NI defines the interface between the NCAP and the network holding other nodes.

#### 2.1.5. Simplified Architecture

**Figure 2:**
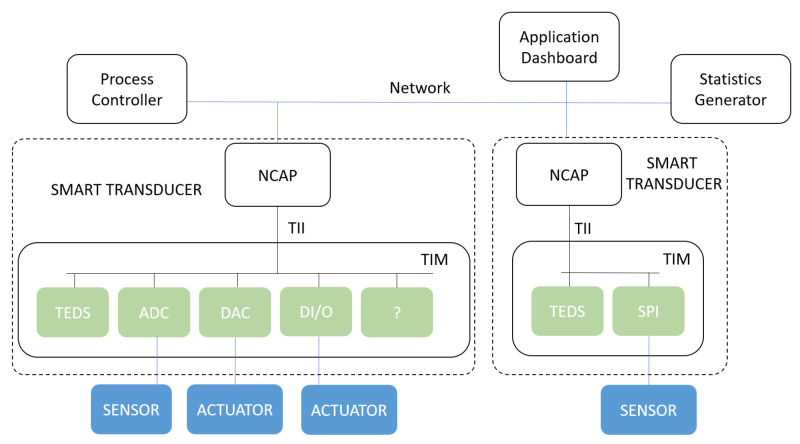
Basic architecture for a distributed control loop based on smart transducers.

In Figure 2, a simplified architecture based on IEEE 1451 is proposed. It presents a simple control loop containing two Smart Transducer Modules and a logical process controller (e.g., PLC). The module on the left presents multiple connections to sensors and actuators, making it behave like a custom I/O card. Such a solution could be implemented to install transducers in isolated parts of a machine. Having the signal conditioning and acquisition closer to the source of the signal reduces signal interference caused by electromagnetic noise present on a plant floor. The transport of digital information is also more robust than analog data. Furthermore, by only using one cable to connect the NCAP to the network, the quantity of cabling is reduced, an ecological and economically desired progress.

The module on the right presents a situation where a simple transducer is connected to the network. However, including an NCAP responsible for this transducer allows a more complex oversight of this component (e.g., condition monitoring on bearing-based motors). Another possibility not depicted in Figure 2 is having multiple TIMs connected to one NCAP as mentioned in Section 2.1.3. The distribution of smart transducers over an edge-component-based network can turn the sampling of sensors into more elaborate functions, allowing the integration of newer techniques to be used in distributed control loops (e.g., machine learning, data mining, condition monitoring). Such is the example included in the figure, including the introduction of an application dashboard and a statistics generator.

#### 2.1.6. Summary

The advantages of using IEEE 1451 are well-cited above: isolation of responsibilities, standardization of interfaces, the modularization of I/O systems, and giving processing power to the edge level of a system. IEEE 1451 also eases the scalability and upgradeability of an automation system by making the nodes easily integrable into the control loop.

Having self-description and self-identification helps the development of control software as it gives the programmer clear and technical instructions about the capabilities of the transducers installed and what they can do. This group of features enables system designers to move one level up the abstraction pyramid. Sensors and actuators no longer need to be thought of as physical components. These are transformed into services that logic controllers and other CPS applications can use.

By abstracting systems developers from the physical implementation of transducers, higher-level concepts can more easily be deployed into networks. Techniques such as machine learning or data analysis also largely benefit from having access to endpoints providing structured data (information) [14].

The use of network-capable nodes also brings advantages to the interoperability of systems. An example of this might be a logical process controller and a condition monitor, using the same sensor concurrently to fulfill their objectives. The openness provided by the distributed implementation facilitates resource sharing.

### 2.2. Sensorization and Actuation Solutions Using IEEE 1451

IEEE 1451 is more present in literature in an application way, meaning, IEEE 1451 is used more as a means of obtaining robust integrated sensors and actuators other than being studied itself. This fact is relevant and important, as it shows this standard is not dead as it is being utilized and refined over time. The current literature review stands on the developments made using the original IEEE 1451 standards, released from 1997 through 2010. It reviews articles in various different applications in order to fetch the most relevant requirements to be implemented in this work.

#### 2.2.1. Review on the Applications of IEEE 1451

IEEE 1451 has been used as a way to implement smart transducers to the most various field of application, extending to all kinds of industries. A literature review was conducted to understand what has been made using the IEEE 1451 standard, the technologies used, and which IEEE 1451 features are more implemented.

In [15], Ulivieri et al. made use of the plug-and-play and self-configuration capabilities of IEEE 1451.4 to create one artificial “nose” to sense different gases. The self-configuration capabilities allowed Ulivieri et al. to create a sensor array in which different resistive chemical sensing sensors can quickly be arranged to ‘smell’ different gases.

In [16], Serra et al. used IEEE 1451 node computing capabilities and the reduction in power usage related to the reduction in transmissions needed for data acquisition to create a wastewater management system. NCAP interoperability features are also used by Serra et al., allowing for direct communication between sensors and actuators deployed in different NCAPs.

Wang et al. developed a network-capable smart sensor [17] that captures the audio from a microphone and used a configurable digital signal processor (DSP) filter to clean the signal obtained from the source. It also uses the communication capabilities of IEEE 1451 to configure the sensor during operation. Furthermore, Wang et al. summarized the IEEE 1451 standards and the specific hardware choices for NCAP, STIM, and TII design.

Making use of the TII interface independence provided by IEEE 1451, Batista et al. implemented a smart instrumentation system based on an FPGA system [18]. Batista et al. also utilized the system adaptability provided by the TEDS to create an easily reconfigurable system. The standard is fully detailed and hardware and software implementations are thoroughly explained. Both wired and wireless versions of IEEE 1451 are studied and deployed.

Utilizing IEEE 1451.5 introduction of Zigbee capabilities, Higuera et al. proposed a one NCAP to many Wireless TIM’s (WTIM) architecture [19], natural of Zigbee implementations. The TEDS was thoroughly analyzed and a proposal was made to improve the TEDS definition for Zigbee. Tests were carried out using one NCAP and three WTIMs verifying the interoperability and plug-and-play characteristic IEEE 1451 introduces.

A smart comfort sensing system [20] was created by Kumar et al. The intent of this system is to estimate the comfort of a building by sensing the presence of different gases, such as Carbon Monoxide and Dioxide, the sensing of temperature of the building, and the sensing of the humidity level. Building on top of IEEE 1451 communication and interoperability capabilities, Kumar et al. developed a Wireless Sensor Network (WSN) responsible for retrieving data from all sensors and carrying out adjustments to the building’s Heating, Ventilation, and Air Conditioning (HVAC) system in order to maximize the air comfort for its occupants.

In [21], Wei et al. utilized IEEE 1451.2 to create a pH measuring system, developing and configuring the TEDS in a way that the calibration adjusts itself based on the temperature of the medium being measured. This capability guarantees the correct pH readout. Wei et al. also utilize an adaptation of IEEE 1451 to allow for the USB connection to the intelligent sensor to carry readout and calibration operations.

Malar et al. [22] devised a smart transducer based on IEEE 1451.4. It uses a mixed-mode interface implemented at the STIM level to capture signals and transmit them to a Digital Acquisition System (DAQ) which functions as the NCAP. The article also presents deficits in the smart transducers industry. It states that, although there are already some manufacturer solutions for smart transducers, there is a lack of development tools for creating such intelligent transducers. Malar et al. proposed an architecture to promote the introduction of self-identification features to existing sensors through the addition of a TEDS-holding microcontroller. To test this hypothesis, two transducer modules were implemented: a load sensor with a measuring bridge and a thermometer based on a thermocouple sensor. Experiences showed success in this implementation. The work Malar et al. started is highly relevant to the presented work. Whilst recognizing the relevance of this work, Malar et al. failed to achieve the ease of smart transducer insertion into a system that the current work intends to get. Moreover, the work presented is not public, meaning, the work developed by Malar et al. also fails to provide tools for the scientific community to build upon, which this work intends on.

**Figure 3:**
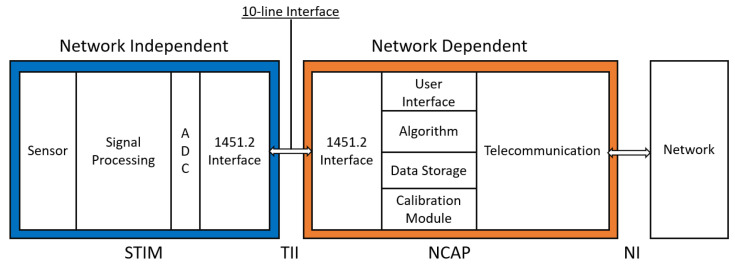
Basic smart transducer architecture (as used in Fan et al.).

Taking IEEE 1451 to a completely different and unexpected application, Pinto et al. created a Vehicular Sensing Network (VSN) [23] based on e-bicycles with the intent to monitor the mobility platform, i.e., the e-bicycles’ battery usage and location, and to measure the air quality throughout a small city. To do this, Pinto et al. defined an infrastructure in which TIMs are installed in bicycles coupled with ozone, humidity, temperature, and energy metering sensors. NCAPs are implemented into the user’s mobile phone which is responsible for monitoring three data transducing systems: the bicycle’s TIM, the phone’s GPS sensor and a virtual TIM implemented as an Android app featuring a user interface. There are also NCAPs installed in the e-bike photovoltaic charging stations. Communication between NCAPs and TIMs uses Bluetooth (IEEE 1451.5 [9]). The TIM, NCAP, and TEDS implementation is explained by Pinto et al. [23], and the authors conclude on the versatility of the solution implemented thanks to the IEEE 1451 standard being used. This versatility is again evidenced in the future work proposed already foreseeing the integration of new sensors into the network (e.g., fall detection sensor). In a more practical approach, Fan et al. implemented a smart transducer [24] based on IEEE 1451.2 [6]. The STIM implementation is made on an FPGA and the NCAP is implemented around an STM32 microcontroller. A detailed report on the work developed is presented, explaining the TII implementation (10-pin SPI interface as per standard). Tests on the network interface are also made, testing CAN, RS-485, SPI and Universal Asynchronous Receiver-Transmitter (UART). This work is concluded by presenting successful reports on the implementation of sensor communication. Whilst this paper introduces no new scientific knowledge, it presents a hands-on approach in the part of the work this is focused on, proving the feasibility of the work. The simple architecture used by Fan et al. is presented in Figure 3.

Relating to the topic of this work, Cherian et al. present work on an IoT interface for industrial analog sensors [25]. An NCAP + TIM integrated solution is developed allowing for the input of four types of sensors: voltage-based, current-based, pulsed outputs, and NPN/PNP or dry contact sensors. For TEDS communication, XMPP is used, as per standard IEEE 21451 [26]. The transducing platform presented is also powered through the network using Power over Ethernet (PoE) which is an interesting approach. Cherian et al. discuss the importance of upgrading functioning analog sensors while giving them processing capabilities, one important topic this work fits right in. The upgrading of a functioning and high-precision manufacturing system is more times than opposite, cheaper than replacing the system with a new one. This work is highly relevant to the present article but, again, there is no access to the tools developed by the authors.

#### 2.2.2. Presented References Analysis

A summary of the works presented above is introduced in this subsection. An analysis is made in Table 1 about the technology components utilized, the industry it is applied to and the type of network used (either wired or wireless).

Table 1 shows the broad applications IEEE 1451 can have. The sheer number of standards published (IEEE 1451.0,1,2,3,4,5,7) can imply this. The necessity of defining multiple possible TII interfaces allows the usage of IEEE 1451 in the most creative and collaborative ways. Everything from a simple microphone filtering system [17] to a small city e-Bicycle management system [23] can be achieved through the IEEE 1451 group of standards.

The most utilized feature of IEEE 1451 is TEDS [27] and its ability to standardize data storage and communication in smart transducers. The plug-and-play concept is also much utilized as it allows for the ease of integration of new transducers into a network.

The literature review conducted showed many application papers utilizing IEEE 1451 to achieve a goal, almost never conducting any analysis on the standard itself. Although there are many application papers on the subject, it was not possible, to the best knowledge of the author, to find tools to develop transducing systems using IEEE 1451. The academic community recognizes the importance of the standard and only utilizes it as a tool to develop systems. A search through Google Scholar, Scopus and Web-of-Science also retrieved many book chapters talking about the importance of smart transducers and pointing to IEEE 1451 as almost an absolute way to achieve them. It can be concluded that, in the industrial area, a smart transducer equates to IEEE 1451.

### 2.3. Final Remarks

From the literature review that has been conducted so far, some conclusions can be drawn:

- IEEE 1451 was born from the industrial market needs and was implanted as the solution to follow to obtain a smart transducer;
- The IEEE 1451 standard still has space to grow into industrial applications. IEEE 1451 still remains at an academic level of use and, while some real use case applications exist, the introduction of a smart transducer module would be relevant;
- The developments made using IEEE 1451 are almost always custom-fitted to the application needed. Work towards universal smart transducing modules would be relevant;
- There is a lack of tools to help the development of transducing systems that can adapt to multiple applications.

This work appears to be relevant in helping the IEEE 1451 standard to be implemented in industrial environments. To the best of the authors’ knowledge, there are no IEEE 1451-based smart transducers commercially available. The only available systems are in the academia 1, but are closed-source. The development of a configurable system to sample non-smart transducers and tools to create smart transducers, according to the literature review above seems fitting and important to allow for the testing and deployment of smart transducer systems into the plant floor. This system can then be used as a tool for testing, deployment and integration of transducer systems. Before this work, there was no widely shared tool to develop IEEE 1451-based smart transducers. Opening this development back to the scientific community also seems important, to allow others to develop and build upon the knowledge this work intends to create. Through the utilization of this system on a larger set of scenarios, limitations and problems can be found and addressed to create a better, more coherent solution.

## 3. Materials and Methods

From the bibliographic review in the previous chapter, no general and open solution was found to contribute to the usage of IEEE 1451 in industrial environments. Moreover, the transition to Industry 4.0 architectures imposes a high demand for meaningful asset information, which motivates the adoption of IEEE 1451. These premises, combined, justify the need to develop a solution that makes the IEEE 1451 easier to use in industrial environments. There is a need to create a system that can handle different use cases related to production scenarios and be easily programmed to fit these production scenarios.

Ease of reconfiguration, modularity, and expandability come as goals to try and meet within this work. This work also lines up with previous and current work being developed by the author, which is detailed next.

Before this work, the author was involved in the “EIT-Manufacturing FactoRIS” research project [28]. Inserted in this project, a learning factory demonstrator was refurbished to serve as a learning media to introduce students to zero defects manufacturing, condition monitoring, and system integration [29]. In [29], the author et al. detail the process behind the refurbishment and upgrading of an old but functional learning factory. Inserted into these developments, there was the need for adding, upgrading, and integrating sensors and actuators into an already functioning system. A large volume of the work included in the DIGI2 laboratory projects is the integration of sensors and the creation of endpoints to fetch data from in order to be handled or stored for future processing. Moreover, many of the problems mentioned regarding sustainability, reparation and upgrading of systems described in the introductory chapter were common to many of the projects developed.

This work is associated with the “Continental FoF” R&D Project. “Continental FoF”’s primary goal is to create technical and academic knowledge to aid in the development of state-of-the-art products that are to be integrated as a way to create a *Factory of the Future*, to be deployed in Continental’s Advanced Antenna (AA) factory. This knowledge ought to be used to address the complex challenges arising from the new technological requirements expected for future-generation vehicles.

As is well known, control, optimization, and prediction techniques are highly dependent on information. In an industrial environment, information is mainly generated by sensors and deployed by actuators. When there is enough benefit to applying some of these techniques, the process behind deploying them includes assessing the information available, and, if required, installing new sensors and actuators. To sustain the development of an industrial cyber-physical system for the *Factory of the Future*, there is a specific need to devise an edge device solution ready for the plug & play of sensors and actuators. The motivation behind this device is the software development and configuration effort currently required for technicians to integrate new transducers. Products, processes, and assets will be equipped with transducers whose information will be used for intelligence purposes. The development of transducer integration techniques motivated this work.

### 3.1. Architectural Goals

The main objective of this work is to develop a platform for converting normal sensors and actuators into smart transducers based on IEEE 1451. It shall be capable of interfacing with any transducer to collect and deploy data, convert it to information and communicate it to a network in a real industrial use case scenario. The development of a smart transducer interface module responsible for sampling and conditioning signals for both digital and analog sensors will achieve this objective.

With such goals and objectives in mind, the following architecture was developed.

### 3.2. IEEE 1451.0 Reference Architecture

**Figure 4:**
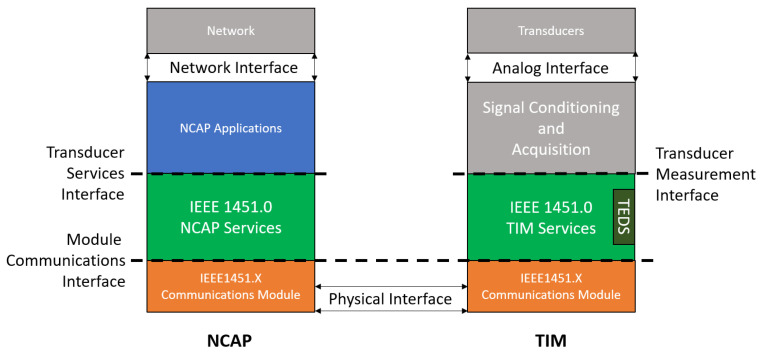
IEEE 1451.0 reference model (adapted from the Standard [4]).

IEEE 1451.0 [4] describes a reference model for a smart transducing system, presented in Figure 4. In this reference model, two main components, the NCAP and the TIM perform complementary actions that communicate via a Communications Module.

The main goal of this work is to develop a transducer interface module and, as such, regarding this reference architecture, is important to point out that:

- The communications module can be either defined through IEEE 1451 standards or implemented through a proprietary module referred to as compliant to fictitious standard IEEE 1451.X. Any communication module complies with IEEE 1451.X as long as it implements the interfaces defined in IEEE 1451.0 referring to the communication module.
- The transducer measurement interface is left for the developing team to define, with no well-defined interface defined. This interface should be designed to allow the recollection of data that allows the TIM IEEE 1451.0 services to be provided.

An IEEE 1451.0-compliant smart transducer system must also implement the transducer services interface on the NCAP side.

### 3.3. Architecture Proposal

Extrapolating from both the IEEE 1451 architecture and the objectives retrieved from the Bibliographic Review, some design objectives were set:

- Connecting multiple TIMs to one NCAP, conceding plug-and-play capabilities to this connection;
- Connecting multiple transducers connected to one TIM;
- Making the addition of transducers to the loop control as easy as possible;
- Making the TIM independent from the signal conditioning circuitry.

**Figure 5:**
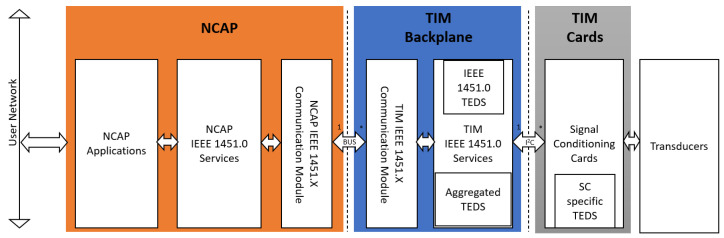
Architecture Model Proposal, built around IEEE 1451.0’s reference model.

With such objectives in mind, the architecture presented in Figure 5 was devised. Following this architecture, the transducing system comprises three main components: the NCAP, the TIM backplane, and the TIM signal acquisition and conditioning cards. As this architecture implements the standard, many of the blocks are similar to the ones found in the reference model. However, this architecture presents some differences, which will be evidenced in this section.

#### 3.3.1. NCAP

The Network Capable Application Processor is responsible for serving as the processing node which coordinates operations inside the IEEE 1451-compliant system. It is responsible for holding applications that make use of the transducer aggregation power provided by the TIM. The NCAP was not explored in this work.

#### 3.3.2. TII (Communications Module)

The Communications module serves as the interface between the NCAP and TIM. Through the communications module, commands and messages are passed from the NCAP to the TIM and in reverse. This layer ought to be utterly opaque to the NCAP and TIM. The communications module must only implement the communications module API functions.

For this specific work, versatility is at a premium, so the ability to connect multiple TIMs to one NCAP is highly relevant. IEEE 1451.0 provides two different approaches for communication modules: point-to-point communication and network communication. According to the wanted applications, network communications were chosen.

Of the physical implementations for networked communication, CAN 2.0 B was chosen. It was the technology that better fitted into the proceedings of the DIGI2 laboratory. Moreover, it presents the following benefits:

- Extended Addressing, allowing both sender and receiver to be identified in the messages being sent;
- Low Latency in small messages, such as simple sensor readouts;
- Error detection and correction at the data-link level;
- Easily deployable peer-to-peer communication;
- Easy scalability of a TIM network.

#### 3.3.3. TIM Backplane

The TIM Backplane represents a physical hardware module that implements and runs the TIM IEEE 1451.0 Services. It is responsible for receiving, decoding, and interpreting the commands from the NCAP, orchestrating communication with the TIM Cards, fetching data from these, and reporting back to the NCAP via a message. Since the TIM Cards also require plug-and-play features, it is also responsible for deploying registration services for TIM Cards and managing the timings for communication with these. It also holds the TIM Meta-TEDS and all configurations common to all transducer channels.

#### 3.3.4. TIM Cards

TIM Cards serve as the physical representation of transducer channels. They carry all the signal conditioning circuitry needed to interact with physical transducers. For this reason, all the configurations and information relative to each transducer channel are also stored inside each TIM Card (encapsulated into each transducer Channel TEDS). It must also contain communication capabilities and be implemented in a way that features plug-and-play capabilities into a $I^{2}C$ bus. TIM Cards should be hot-swappable, meaning they can be inserted or removed from the system without disrupting its normal functioning. Each TIM Card necessarily has to feature a microcontroller to implement and convey all the functions required for the correct functioning. This card might encapsulate multiple sensors and mask them as one or carry out simple operations such as comparative or signal amplification.

#### 3.3.5. Backplane to Card Interface

The interface between the cards and the backplane shall be implemented using $I^{2}C$, featuring hot-swappable buffers in order to allow for electric compatibility with plug-and-play behavior. The backplane to card interface must also carry power to the cards to reduce wiring and centralize power distribution. If the card needs external power, the external power will not be utilized.

#### 3.3.6. Advantages of the Transducing System Architecture

The architecture hereby presented attains new degrees of flexibility in transducing systems. By transforming the TIM into a mutable and adaptable component of the system, the objective is to provide ease of upgradeability, expansion, repair, or substitution of components. Moreover, by containing all the signal acquisition circuitry inside a card, the TIM Backplane can be reutilized between different projects or upgraded inside the same project without the need to replace all the signal acquisition circuitry.

Imagine the following scenario: by the proposed architecture, the TIM backplane can become a bottleneck in the polling frequency of the system (if, for example, there is the need to sample faster for a specific process). To solve this problem a TIM backplane based on a faster processor can be easily swapped in, or the load might be distributed against multiple TIM backplanes, requiring little to any system configurations. With this approach, the TIM backplane becomes a simple component of a broader ecosystem, all interconnected with information being absorbed internally from the TIM Cards.

In the same manner, the TIM Cards are also expected to bring more flexibility to the system. Imagine having a custom sensor that needs specific hardware to read its measurement (e.g., a load cell measured through a Wheatstone bridge). Once the card is custom fitted around this sensor, it can be deployed into each and any TIM backplane. This can become an advantage if sampling hardware needs to be reused in different scenarios and reutilized over time. A temperature sensor removed from a functional asset no longer needs to be discarded as long as it is kept coupled to its card. It will become just another asset waiting to be deployed into a new system. This contributes to reducing electronic waste and adds to the sustainability of the system proposed.

**Figure 6:**
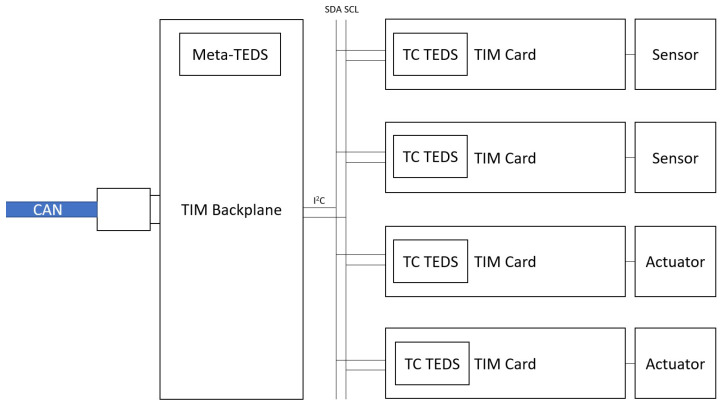
Graphical representation of an implementation of the presented architecture.

A simplified visual diagram of the hardware interconnections can be seen in Figure 6.

Implementing the TIM along with a robust and well-tested interface such as CAN is expected to provide a strong industrial backbone, allowing for the distribution of TIMs in networks in order to provide a multi-drop transducing system. This approach allows distributed measuring and actuation systems to be more easily deployed on the shop floor.

Introducing the TIM Cards is also expected to bring novelty and flexibility to the traditional TIM approaches, sometimes seen in literature as a One TIM-One Sensor implementation. This approach, even if theoretical, expects to be a basis for future work.

### 3.4. Implementation Platform

The nature of the problem being solved, developing a transducer interfacing system, carries along the development of software and hardware to fulfill communication-bus-depending, speed, and physical transducer interfacing requirements. The specific case described here fits into the realm of embedded systems, with the customization of both hardware and software to fit a clear and well-defined purpose.

This type of system, with such proximity to the hardware level, called for a microcontroller system designed with communication and general-purpose interfacing in mind.

#### 3.4.1. Microcontroller: TM4C123XL Platform

**Figure 7:**
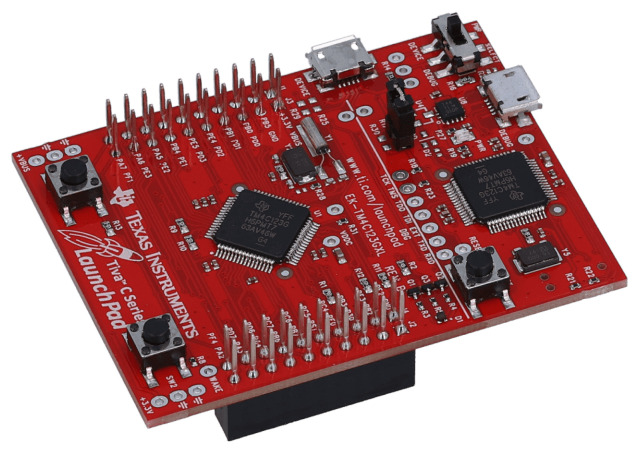
Texas Instruments TM4C123GXL Development Platform.

The TIM was developed around the TM4C123GXL platform (represented in Figure 7). The TM4C123GXL is a development platform based on the TM4C123GH6PM microcontroller by Texas Instruments. The TM4C123GH6PM is designed around an ARM Cortex-M Processing core running at 80 MHz. It features a 256 kB Flash Memory, 32 kB of RAM, and 2 kB of EEPROM, 8 Universal Asynchronous Receivers/Transmitter (UART), 4 Synchronous Serial Interfaces (SSI), four high-speed Inter-Integrated Circuit (*I*^2^*C*) modules, 1 Universal Serial Bus (USB) 2.0 interface, and 2 Controller Area Network (CAN) controllers. For signal acquisition and control, it features 6 General Purpose Input Output (GPIO) blocks, 16 Pulsed Width Modulation (PWM), and two 12-bit Analog to Digital Converters (ADC).

In order to facilitate and potentiate software development, the software was devised over the TI-Real-Time Operating System (TI-RTOS) [30].

To allow the interface with the CAN bus, a prototype shield was devised.

#### 3.4.2. Development Shield

**Figure 8:**
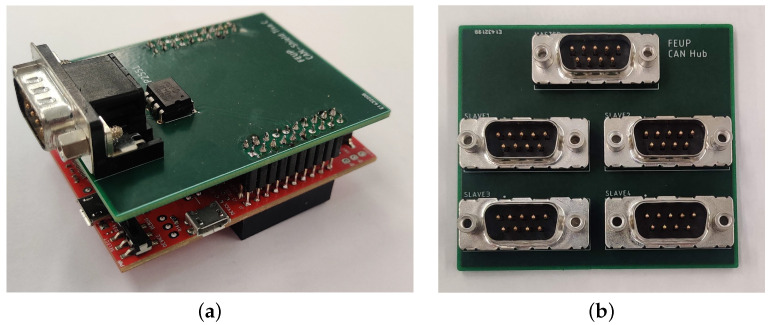
Final PCBs: (**a**) CAN shield PCB assembled; (**b**) CAN hub PCB assembled.

As referred to previously, there is a need to communicate with a CAN bus. Although the microcontroller features CAN bus controllers, it requires a CAN bus transceiver to act as the interface between the 12 V logic of the CAN bus and the 5 V logic from the microcontroller. With such intent, the integrated circuit MCP2551, a high-speed CAN transceiver, was introduced to the circuit. For this, a printed circuit board was projected in order to have a more sturdy and reliable solution. In parallel with the CAN shield, a CAN Hub was also designed in order to facilitate connections from one NCAP to many TIMs all in the same CAN bus. The resultant boards can be seen in Figure 8a,b.

The PCBs fulfilled their main objective: providing a solid and robust base to work on.

## 4. Implementation-Design and Structure

IEEE 1451.0 presents well-defined interfaces through the module communications API presented in chapter 9 of [4]. In developing the software platform for the STIM, the module communications API was used as the primary source to define the software architecture. Around it, two main modules were developed: the IEEE 1451.0 TIM services and the IEEE 1451.X Communication Module, based on CAN.

There is a crucial aspect to be mentioned: the interface between TIM Cards and the TIM backplane. Although initially planned, and the fact that the architecture presented TIM Cards, these are not employed in the current state of the work. Timing and resource limitations led the work to merge TIM Cards and backplanes into a TIM Module. This merge does not mean the architecture is irrelevant; it will only be left as future work as the other blocks of the system are fully ready to accept this change.

### 4.1. IEEE 1451.0 Standard-System Specification

**Figure 9:**
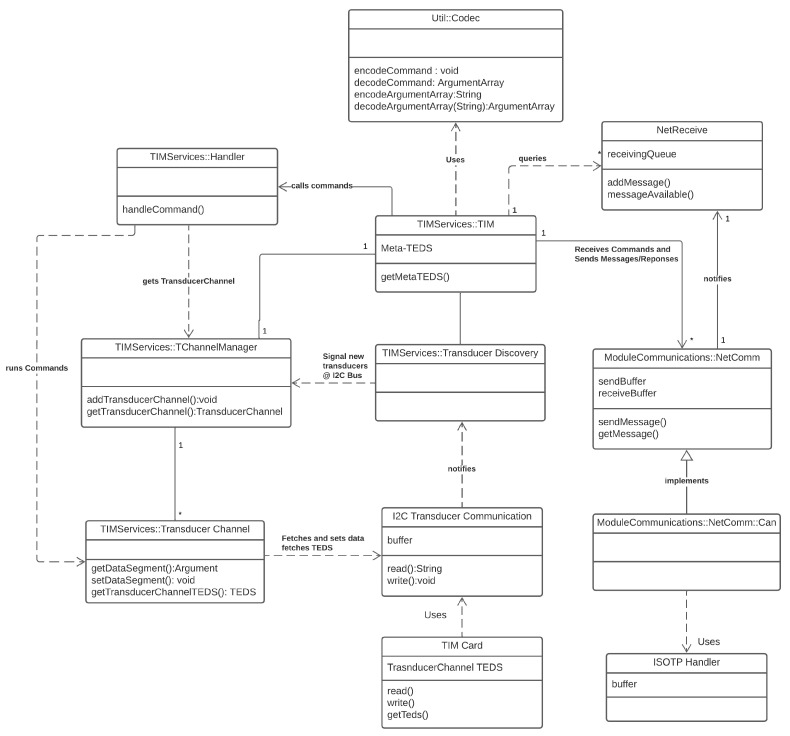
TIM architecture implementation UML class diagram.

Following the architecture proposed in Section 3.3, the diagram presented in Figure 9 depicts a top-level structure of the software produced to implement the standard.

### 4.2. TIM Services Module

The TIM services module is the module that defines and implements the necessary classes and interfaces to allow for the implementation of the STIM functional model depicted by the IEEE 1451.0 standard. The standard does not strictly define the TIM services module, so its structural architecture is left for the developing members to define.

The main mechanisms behind the functional model are transducer channels. A different transducer channel represents each physical transducer and encapsulates all the functionalities needed for the operation and interaction with the said transducer. According to the object-oriented paradigm, each transducer channel behaves like a class and defines the transducer channel class. The transducer channel manager class is responsible for storing and modifying a list of all transducer channels attached to the TIM at a given time.

The top class in the TIM Services Module is the TIM class. The TIM class holds all methods and attributes relative to the TIM or common to all transducer channels. Such is the case of the Meta-TEDS, which is stored and updated through the TIM class. The TIM class also handles the commands received by the TIM through the handler class. If the command is related to the TIM itself, the TIM object handles it directly. If the command is tailored towards a transducer channel, the handler fetches the TC from the transducer channel manager and performs the actions directly on the transducer channel. Actions (commands) over a transducer channel include but are not limited to:

- Obtain and set the transducer channel TEDS;
- Obtain a reading from a sensor;
- Configure the transducer channel way of polling the sensor;
- Trigger a buffered read;
- Set the output of an actuator;
- Configure how an actuator might be triggered;
- Obtain the Status of the transducer channel.

Each of the actions above is mapped to individual commands which carry along arguments, passed through the Argument Array concept described above. As some commands require the TIM to respond, if that is the case, the TIM will handle the command and generate a response which will, in turn, be encoded and sent, via the communications module, back to the NCAP.

### 4.3. Summary

There are some missing features to make the TIM fully IEEE 1451.0 compliant, but the features implemented at the time of writing this article follow the standard. This makes the TIM IEEE 1451.0 compliant to some extent. There is also the TIM Cards feature which is not implemented but will be in future work.

All the software developed here is released as open-source to allow for modification, application, and improvement. The way the firmware is built intends to facilitate the introduction of new implementations of the interfaces using dependency injection. Using the developed and presented architecture is also expected to help further developments in the future time.

## 5. Sensor and Actuator Integration Results

The smart transducing system intends to be integrated into industrial environments. Test scenarios were devised to show the different approaches that can be taken while using the developed platform.

### 5.1. Working Examples

Some simple scenarios were set to verify the system’s correct functioning. These scenarios pretend to illustrate the plug-and-play capability of the system recognizing the TIMs installed in the network and the sensors and actuators that are transducer channels. For this example, temperature sensors were used as the transducer to be read.

There are three scenarios presented:

- One NCAP, One TIM, One Sensor;
- One NCAP, One TIM, Four Sensors;
- One NCAP, Four TIMs, One Sensor per TIM.

Each of these approaches will be described and characterized in the following subsections.

#### 5.1.1. One NCAP-to-One TIM-to-One Sensor

The scenario hereby presented is the most simple one. There is only TIM in the system, which only contains one sensor. This replicates a typical networked sensor but is implemented using IEEE 1451.0. For example, one could argue that this setup resembles the IEEE 1451.2 TII definition, only exchanging the 10-wire interface for a CAN-based communication system. For this and the following examples, a simple SPI temperature sensor based around the MAX31855 Thermocouple-to-Digital converter was used [31]. It communicates via an SPI interface that outputs the temperature readout along with some flags once triggered. The transducer channel is capable of decoding the temperature from the readout and outputting the temperature in Celsius. It could, for example, be used in a simple temperature monitoring system in a greenhouse.

**Figure 10:**
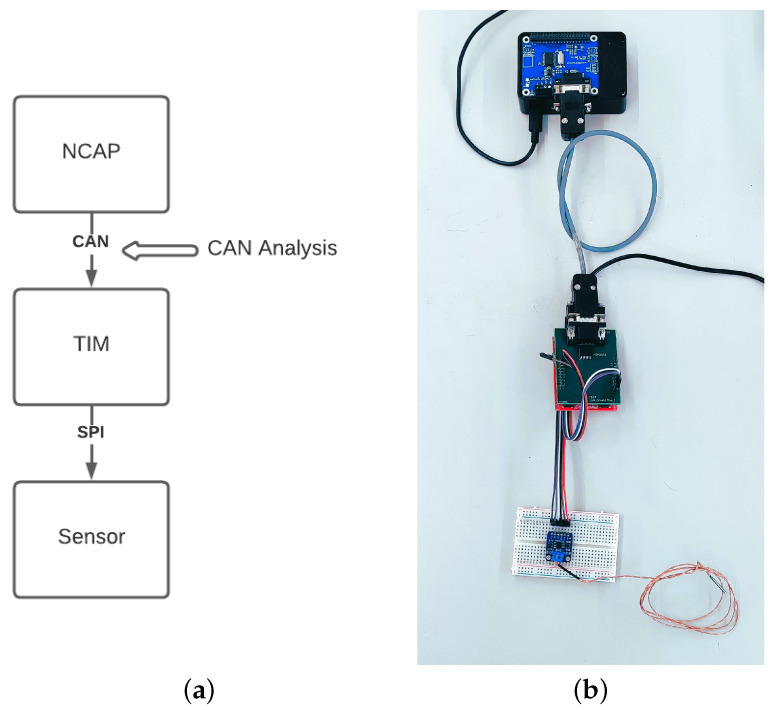
One-to-One-to-One System: (**a**) Diagram representing one-to-one-to-one system connection; (**b**) Physical representation of the connected one-to-one-to-one system.

A basic diagram and setup pictures are provided in Figure 10. Please note this setup does not make use of the CAN Hub.

#### Connection

When the system starts up, it begins by executing the handshake protocol with the NCAP. During the handshake, the NCAP provides the TIM with a communication ID. This is followed by a Meta-TEDS request by the NCAP. The TIM responds with the Meta-TEDS, which carries the information that the TIM currently holds one transducer channel. With this information, the NCAP requests the transducer channel TEDS to know what can be sampled from the transducer channel and the TIM responds. In this case, the temperature can be sampled. The system is now fully described and ready to start sampling. In order to fully comprehend the communication process that goes behind the connection and registration process, the specific CAN frames carried over the CAN BUS are presented and explained in Appendix A.

#### Sampling

The transducer channel inserted into the presented system is configured into immediate sampling mode. This means when the NCAP sends a command requesting the Transducer Channel Data Segment, the TIM will generate a request for the transducer channel to go ahead and sample the sensor. After the sensor is sampled, the TIM generates the response and sends it to the NCAP, carrying with it a success flag. If the operation cannot be carried out, the success flag will not be set. Again, the specific CAN frames for the operation are presented in Appendix A.

#### 5.1.2. One NCAP-to-One TIM-to-Many Sensors

**Figure 11:**
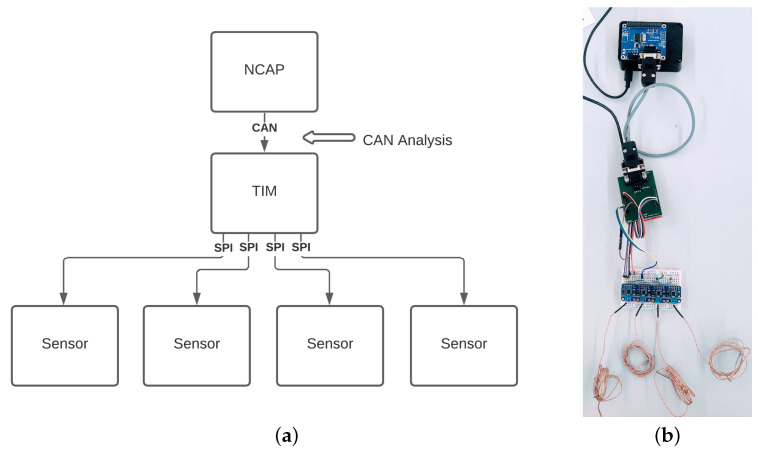
One-to-One-to-Many System: (**a**) Diagram representing one-to-one-to-many system connection; (**b**) Physical representation of the connected one-to-one-to-many system.

This scenario, depicted in Figure 11, intends to demonstrate multiple transducer channels inserted into one system. As the example still uses temperature sensors, it could equate, for example, to a temperature readout in a robotic arm, sampling the temperature of different motors. The primary behavioral difference, in this case, is the sampling of different transducer channels. This will equate to the change in the Meta-TEDS number of transducer channels and will enable the TIM to sample multiple transducer channels.

Please note that whilst the system configuration changed quite a lot, the architecture allows the system to operate with few updates to the TEDS. At this moment, the SPI channels must be manually introduced into the software, and, still, the implementation effort is minimal. This is all thanks to the flexibility allowed by the software architecture and implementation.

#### 5.1.3. One NCAP-to-Many TIMs-to-One Sensor

**Figure 12:**
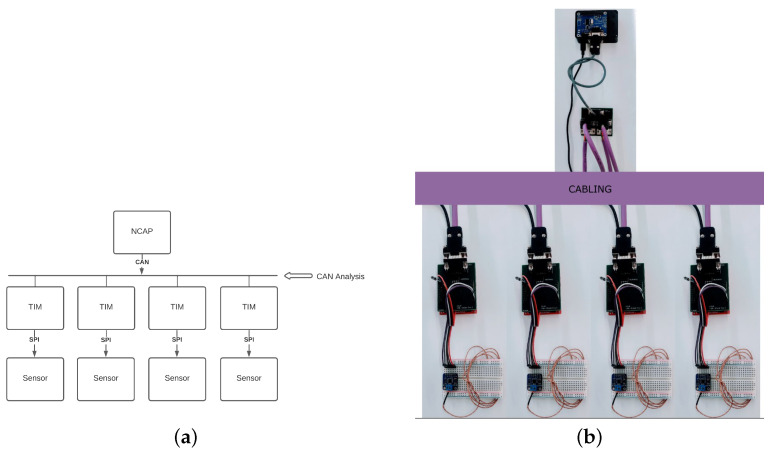
One-to-Many-to-One System: (**a**) Diagram representing one-to-many-to-one system connection; (**b**) Physical representation of the connected one-to-many-to-one system.

The last scenario, depicted in Figure 12 to be presented here is one where the temperature sensors are distributed amongst different TIMs in the network. This scenario intends to demonstrate the ease of deployability of the system in a distributed scenario. It is as simple as connecting the TIMs to the network. No further configuration is required. The CAN Hub is required but only because of how the CAN bus is physically implemented.

A great example of a real use case under this scenario would be a drying paint oven in a car manufacturing factory. The temperature sensors could be deployed over great distances (the current spec allows for 100 m according to Table 1 of [32]. This would allow for the sampling of the temperature gradient over distances, along with providing data for data-analysis processes and allowing for the data that is retrieved to be fed into the control loop of the process.

**Note:** The current system can be utilized with up to 253 unique TIM IDs. Theoretically, each TIM would be capable of holding up to 32,767 unique transducer channels. This would mean millions of transducers per NCAP, which strongly demonstrates the flexibility of the architecture. This means the flexibility is not limited by the architecture, but by the hardware limitations. Regarding the EEPROM memory limitations, META-TEDS size and the transducer channel TEDS Size, the system could support up to 16 transducer channels. These limitations can be easily surpassed with hardware upgrades.

### 5.2. TEDS Measurements

Physical and implementation-related limitations bound the operation of the TIMs. Such is the case for the timings for interacting with transducer channels. These timing-related limitations are described using the transducer channels. In order to facilitate comprehension, consolidate the concepts utilized and measure the system performance, timing measurements were taken in order to define the following TEDS fields:

- Transducer channel sampling period/update time;
- Transducer channel read delay time.

As the fields are directly defined by the IEEE 1451.0 standard, each subsection contains the definition per-standard [4]. The sensor characterized is the same one used in the system described in the previous section.

#### 5.2.1. Transducer Channel Update Time/Sampling Period

In light of the implementation that is currently being tested, the author is presented with an SPI-dependent communication sensor readout. As that is the case, the sampling time is defined as the timing difference between the data request through SPI and the full reception of data. The data retrieved from these measurements contained a really consistent timing measurement with 96 measurements of 56 ms and four measures of 57 ms. Considering the worst measurement, this equates to a maximum frequency for polling of around 17 kHz.

These parameters should be set as **0.000057** s.

#### 5.2.2. Transducer Channel Read Delay Time

This time accounts for the overhead between the reception of a command, the decoding of the command, the command handling, transducer channel command execution, response generation, and response encoding. As some of these points are divided into different tasks (with one of them only being executed every millisecond), it is expected for it to take upwards of 2 ms.

For this measurement, the following test was implemented:

1. The NCAP sends a read command;
2. The TIM saves the time the command has fully been received;
3. The TIM executes the command;
4. The TIM saves the time it sends the response.

**Figure 13:**
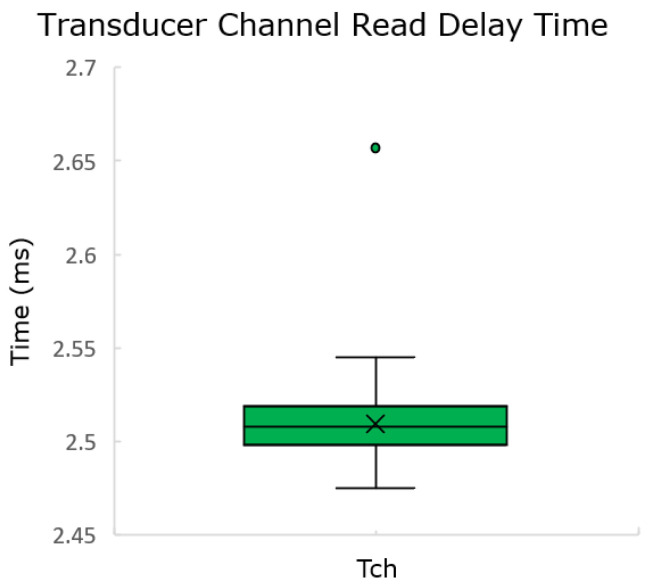
Transducer Channel read delay time measurement.

After 100 measurements were taken, the following boxplot was generated in Figure 13.

The average time for the read delay was 2.51 ms, with the maximum being 2.66 ms. This information tells that, considering the worst case, the system (without communication) can be probed about 250 times per second. This time could be improved, but it would limit the future work on buffered reads, i.e., the capability of the system reading without a trigger.

This field would be set with a value never inferior to **0.0027** s.

**Note:** The previous measurements were taken considering one TIM with only one sensor. Adding more transducer channels will imply a greater load in the system, implying, naturally, worse timing conditions

### 5.3. Handshake Timing Analysis

One of the biggest features of the work here presented is the capability of self-registration and self-identification in the CAN network. These capabilities are even more relevant if they are fast enough that they go unnoticed. The time between **plug** and **play** defines the relevance of this term.

There are two ways of handshake proposed by this work. The TIM can announce itself with a random number and receive an ID from the NCAP or, if the TIM was already given an ID previously, the NCAP can send a discovery command to which the TIM shall respond.

#### 5.3.1. TIM Announcement

It is known that the time for the handshake will be dependent on how many TIMs are present in the network. However, this is not a factor responsible as the TIM is the initiator of the request to identify. Still, the measurement of a simple handshake in a one-to-one configuration seems relevant.

**Figure 14:**
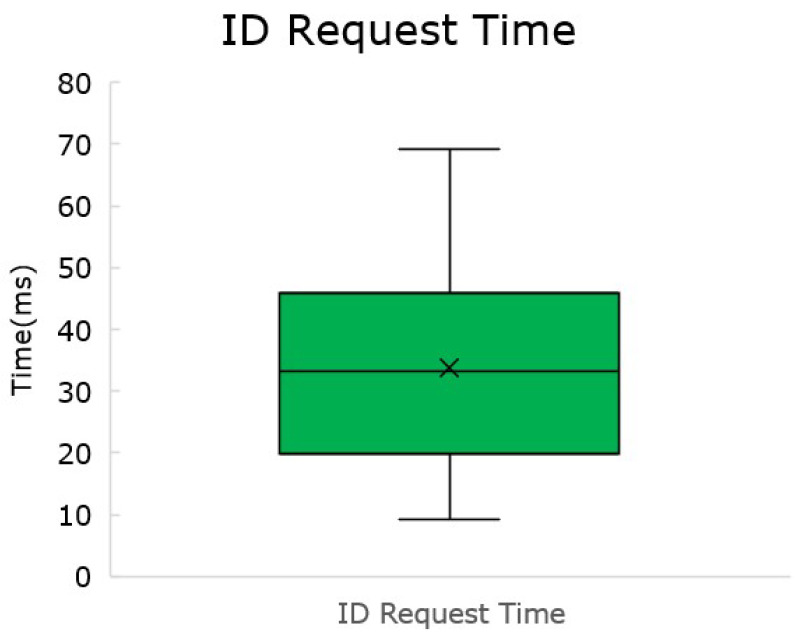
Time for TIM-initiated handshake (accounting for communication).

The time measurement is made from before sending the request for ID to the moment the TIM receives an ID. A boxplot, presented in Figure 14, was generated from 100 measures. The average TIM announcement protocol execution took 33.76 ms.

#### 5.3.2. TIM Discovery

The TIM discovery process functions as a response to a command by the NCAP. While measuring the time it takes for the system to be discovered it is not dependent on the TIM implementation, but on the whole system instead. It is noted, however, that a response to the NCAP discovery command shall take no longer than the TIM announcement protocol.

### 5.4. TEDS Readout Timings

The capability of fetching the TEDS is vital to the functioning of the system. The time this process takes directly influences the time for integration of a new TIM into a network. To discover how much time a TEDS readout takes, two different scenarios were set: the readout of a Meta-TEDS and the readout of a transducer channel TEDS.

#### 5.4.1. Meta-TEDS

The time for reading a Meta-TEDS was measured from the CAN network analyzer and is set from the first frame of the TEDS request command to the last frame of the response.

**Figure 15:**
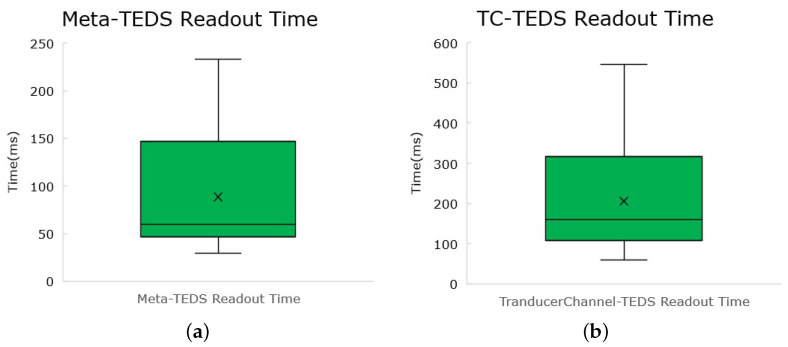
Time for TEDS readout (accounting for communication). (**a**) Meta-TEDS. (**b**) Transducer Channel-TEDS.

100 measures were taken, from which a boxplot was graphed, which can be seen in Figure 15a. The average value of a Meta-TEDS request was measured at **88.5** ms, for a Meta-TEDS of 55 bytes.

#### 5.4.2. Transducer Channel TEDS

The test protocol follows the Meta-TEDS readout time protocol presented above.

100 measures were taken, from which a boxplot was graphed, which can be seen in Figure 15b. The average value of a transducer channel-TEDS request was measured at **205.6** ms, for a transducer channel-TEDS of 114 bytes.

### 5.5. Integration Timing-Methodology Proposal

The integration timing is considered to be measured from the moment the TIM is connected to the network (or is powered up) to the moment the NCAP receives the last transducer channel TEDS. This integration timing is mainly affected by five parameters: the number of TIMs in a network, the handshake time, the time for a META-TEDS request command, the number of transducer channels in a TIM, and the time each transducer channel TEDS request command would take to be handled.

Considering all these factors, a formula was drafted to estimate the time for integration of a smart transducer system, presented below, in Equation (1).

(1) $$t_{integration}={\#}_{TIMS}\times \left(t_{handshake}+t_{META\_TEDS}\right)+\sum_{i=0}^{{\#}_{TIMS}}\left({\#}_{TransducerChannels_{i}}\times t_{TC\_TEDS}\right)$$

Considering the cases presented above, estimations were made for the time for integration of the three different cases. Please take as reference the average captured times of $t_{handshake}=33.76$ ms, $t_{META\_TEDS}=88.5$ ms and $t_{TC\_TEDS}=205.6$ ms. The results for the different cases were computed and are presented in Table 2.

**Figure 16:**
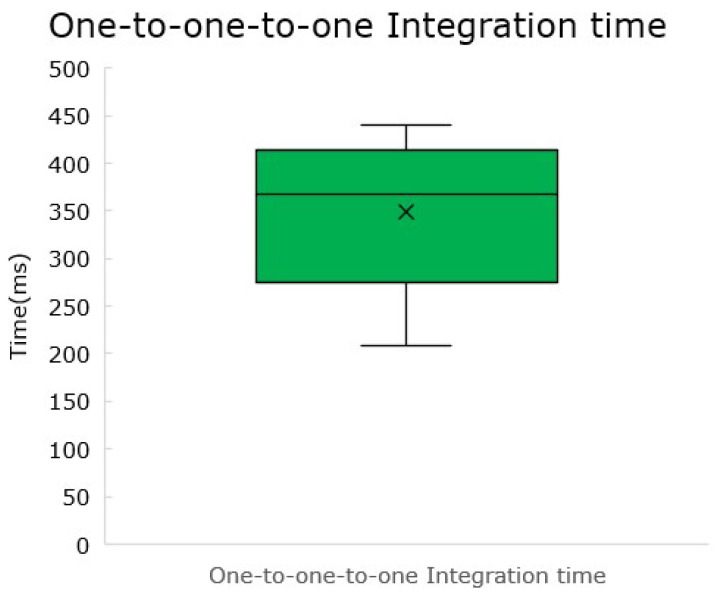
Integration time of the one-to-one-to-one system.

To verify the results in the table above, a verification test was made using the one-to-one-to-one system. It resulted in the times presented in Figure 16.

The results average at an integration time of **348.8** ms, against the 327.8 ms suggested by the estimation formula. The difference between expected and measured is about **6%** and is attributed to the variation of the communication timings, which reflects a large variance from sample to sample.

The formula, while only standing for a loose estimation, is capable of giving the system integrator a tool to figure out how much time the integration of the system will take.

Please note that the integration times account for the hot insertion of the TIM in the system, i.e., without turning the system on and off, which is typical of connecting a sensor or actuator to the system. Being able to introduce a transducer in a system and have it ready to read in a matter of seconds poses a big advantage for system integrators, upgraders, or maintainers.

## 6. Summary and Conclusions

Before the development work, a literature review was conducted. From the literature review, it was concluded that there was no general and open solution for the usage of IEEE 1451 in industrial environments. Aligning the motivation with the literature review, an IEEE 1451-based architecture was presented. This architecture aims to increase flexibility through the encapsulation of hardware, software, and plug-and-play features. It presents a tripartition of the TIM into TIM Backplane, TIM Cards, and the Backplane-to-Cards Interface. This architecture is also based on an NCAP-to-TIM Interface based on CAN. While this architecture was not fully implemented, its design decisions are still deemed relevant and motivate future work in this implementation.

After this, the implementation of a compressed version of the architecture presented is described. Some of the main features of the system are described, along with implementation details that were considered relevant. Work on two main modules is described, the communications module based on CAN (which was not found in the literature) and the TIM services module (also developed from the ground up). This development work tried, at its best effort, to follow the architecture presented, to both facilitate comprehension of the codebase and allow for other applications to reutilize these same developments. This platform is to be released as an open-source solution, to contribute back to the scientific community, especially those who want to develop in the realm of IEEE 1451 systems.

The results chapter presents examples and metrics obtained from utilizing the system. Three use cases were defined and characterized to showcase the flexibility of the applications of the system. The TEDS field-filling process was also showcased, whilst also serving as a performance metering process. The handshake process was also analyzed since it is the basis for the plug-and-play functionality. Lastly, an integration timing metric is defined and an estimation formula is set to help others compare its work with the one here presented. This is expected to help in the scientific process behind IEEE 1451 systems development. The formula was applied to the three use cases presented and compared with a test for one of the cases, achieving satisfying results.

Concluding, the work presented achieved, to a large extent, the objectives initially set:

- A platform for developing smart transducers was devised, achieving interesting results.
- The flexibility of the system demonstrated the fulfillment of the objective of developing a smart and flexible transducer system, while only partially implementing the original architecture.
- The encapsulation, standardization of data, loose coupling, information flow, and plug-and-play features of this system make it relevant and applicable under the new Industry 4.0 techniques.
- This platform is released as an open-source solution.
- An integration timing estimation formula is introduced to motivate comparability between works.

This work hopes to serve as a tool for introducing cutting-edge technologies into the shop floor.

## 7. Resources

The developed resources can be found in https://github.com/jmotp/STIM_1451 (accessed on 21 Septembet 2022).

## Figures and Tables

**Table 1:** Summary on the usage of IEEE 1451.

Reference	Technology Components Used	Industry	Connectivity
Gas sensor (nose) [15]	Plug-and-play, self-calibration	Gas Sensing	Wired
Wastewater treatment [16]	edge processing and interoperability	Wastewater	Wireless
Microphone [17]	Edge processing and TEDS to save sensor characteristics	Audio	Wired
FPGA process automation [18]	Interface independence and TEDS reconfigurability	Automation	Wired and Wireless
Zigbee Study [19]	Zigbee TII, Interoperability and TEDS	Academic Research	Wireless
Smart Comfort Sensing System [20]	Interoperability, wireless communication & TEDS	(Building) Automation	Wireless
pH sensor [21]	Self-calibration through Calibration TEDS, Adaptation to USB	(Fish) Farming	Wired
Smart Transducers for Industrial Automation [22]	Mixed Mode Interface, TEDS and plug-and-play	Industrial automation	Wired
e-Bike Monitoring System [23]	Bluetooth TII, edge computing and TEDS	Vehicular Sensing Networks	Wireless
Smart Transducer [24]	TEDS and 10-line TII	Proof-of-Concept	Wired
IoT Interface for Industrial Analog Sensor [25]	TEDS and Ethernet TII	Industrial Internet of Things	Wired

**Table 2:** Estimation of Integration Time for Different Scenarios.

	Case One-to-One-to-One	Case One-to-One-to-Many	Case One-to-Many-to-One
#TIMs	1	1	4
#TC/TIM	1	4	1
$t_{integration}$	327.8 ms	944.6 ms	1311.2 ms

**Figure A1:**
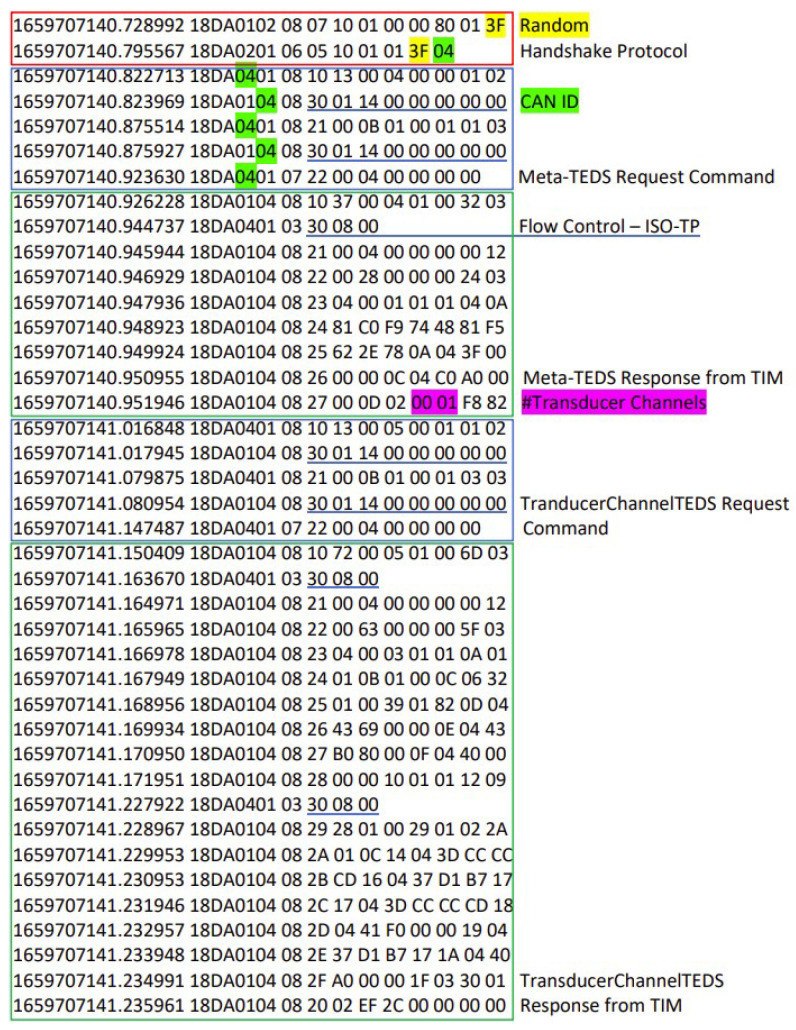
CAN Messages Sequence Between TIM and NCAP.

**Figure A2:**
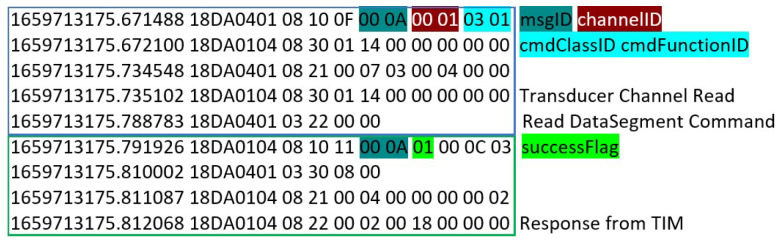
CAN Messages for Transducer Channel DataSegment Readout.
